# Association between genetic variants of the cholinergic system and postoperative delirium and cognitive dysfunction in elderly patients

**DOI:** 10.1186/s12920-021-01071-1

**Published:** 2021-10-21

**Authors:** Maria Heinrich, Miriam Sieg, Jochen Kruppa, Peter Nürnberg, Peter H. Schreier, Stefanie Heilmann-Heimbach, Per Hoffmann, Markus M. Nöthen, Jürgen Janke, Tobias Pischon, Arjen J. C. Slooter, Georg Winterer, Claudia D. Spies

**Affiliations:** 1 Universitätsmedizin Berlin, corporate member of Freie Universität Berlin and Humboldt-Universität zu Berlin, Department of Anesthesiology and Operative Intensive Care Medicine (CCM, CVK), Berlin, Germany; 2 Berlin Institute of Health at Charité – Universitätsmedizin Berlin, Charitéplatz 1, 10117 Berlin, Germany; 3 Charité – Universitätsmedizin Berlin, corporate member of Freie Universität Berlin, Humboldt-Universität zu Berlin, and Berlin Institute of Health, Institute of Medical Informatics, Charitéplatz 1, 10117 Berlin, Germany; 4 QUEST Center for Transforming Biomedical Research, Berlin Institute of Health, Berlin, Germany; 5 Institute of Genetics, University of Cologne, Cologne, Germany; 6 Atlas Biolabs GmbH, Berlin, Germany; 7 Pharmaimage Biomarker Solutions GmbH, Berlin, Germany; 8 Institute of Human Genetics, School of Medicine and University Hospital Bonn, University of Bonn, Bonn, Germany; 9 Human Genomics Research Group, Department of Biomedicine, University of Basel, Basel, Switzerland; 10 MDC/BIH Biobank, Max Delbrück Center for Molecular Medicine in the Helmholtz Association (MDC), Berlin, Germany; 11 Molecular Epidemiology Research Group, Max-Delbrück Center for Molecular Medicine in the Helmholtz Association (MDC), Berlin, Germany; 12 Charité – Universitätsmedizin Berlin, Corporate Member of Freie Universität Berlin, Humboldt-Universität zu Berlin, Berlin, Germany; 13 Department of Intensive Care Medicine, University Medical Center Utrecht, Utrecht University, Utrecht, the Netherlands; 14 UMC Utrecht Brain Center, University Medical Center Utrecht, Utrecht University, Utrecht, the Netherlands; 15 Department of Neurology, UZ Brussel, Vrije Universiteit Brussel, Brussels, Belgium; 16 Charité – Universitätsmedizin Berlin, Corporate Member of Freie Universität Berlin and Humboldt-Universität zu Berlin, ECRC Experimental and Clinical Research Center, Lindenberger Weg 80, 13125 Berlin, Germany

**Keywords:** CHRM2, CHRM4, Genome-wide association study, Neurocognitive disorder

## Abstract

**Background:**

Postoperative delirium (POD) and postoperative cognitive dysfunction (POCD) are frequent and serious complications after surgery. We aim to investigate the association between genetic variants in cholinergic candidate genes according to the Kyoto encyclopedia of genes and genomes - pathway: cholinergic neurotransmission with the development of POD or POCD in elderly patients.

**Methods:**

This analysis is part of the European BioCog project (www.biocog.eu), a prospective multicenter observational study with elderly surgical patients. Patients with a Mini-Mental-State-Examination score ≤ 23 points were excluded. POD was assessed up to seven days after surgery using the Nursing Delirium Screening Scale, Confusion Assessment Method and a patient chart review. POCD was assessed three months after surgery with a neuropsychological test battery. Genotyping was performed on the Illumina Infinium Global Screening Array. Associations with POD and POCD were analyzed using logistic regression analysis, adjusted for age, comorbidities and duration of anesthesia (for POCD analysis additionally for education). Odds ratios (OR) refer to minor allele counts (0, 1, 2).

**Results:**

745 patients could be included in the POD analysis, and 452 in the POCD analysis. The rate of POD within this group was 20.8% (155 patients), and the rate of POCD was 10.2% (46 patients). In a candidate gene approach three genetic variants of the cholinergic genes CHRM2 and CHRM4 were associated with POD (OR [95% confidence interval], rs8191992: 0.61[0.46; 0.80]; rs8191992: 1.60[1.22; 2.09]; rs2067482: 1.64[1.10; 2.44]). No associations were found for POCD.

**Conclusions:**

We found an association between genetic variants of CHRM2 and CHRM4 and POD. Further studies are needed to investigate whether disturbances in acetylcholine release and synaptic plasticity are involved in the development of POD.

*Trial registration*: ClinicalTrials.gov: NCT02265263.

**Supplementary Information:**

The online version contains supplementary material available at 10.1186/s12920-021-01071-1.

## Introduction

Postoperative delirium (POD) is a common and serious complication, presenting as an acute change in the mental state in terms of attention and other cognitive functions [1]. The condition has been associated with increased morbidity and mortality rates [2, 3], as well as with a chronic deterioration of cognitive capacity, termed postoperative cognitive dysfunction (POCD), which can lead to dementia [4, 5].

The incidence of POD and POCD is related to a number of predisposing and precipitating risk factors [6, 7], and although the condition can develop at any age, older patients are particularly susceptible. It is unknown to what extent POD and POCD are subject to genetic factors [8]. However, results of a systematic review suggest that genetic factors are likely to influence the development of POD and POCD [9, 10]. Neurotransmitter imbalance, in addition to neuroinflammation is one of the leading hypotheses for the development of delirium [11]. It was hypothesized that neuroinflammation is driven by a disrupted cholinergic neurotransmission [12]. However, the factors responsible for the disturbance of cholinergic neurotransmission are not sufficiently understood. Genome-wide association studies (GWAS) have so far been unable to identify a link between the cholinergic system and the development of POD and POCD [13, 14], although numerous polymorphisms are located in the cholinergic system [15] and a connection to inflammation has already been established [16]. To confirm the evidence so far of the two previous GWAS, we first performed a GWAS.

Futhermore, the objective of this study was to investigate whether genetic variants in the cholinergic candidate genes were associated with the development of POD or POCD in elderly patients. For that reason we additionally conducted a candidate gene association study (CGAS). Since there is no evidence in the literature indicating which cholinergic genes or variants could be responsible for the disturbance of cholinergic neurotransmission, we decided to investigate all cholinergic genes involved in cholinergic neurotransmission according to the Kyoto encyclopedia of genes and genomes (KEGG) pathway [17–19].

## Methods

### Study design and population

This analysis was conducted as part of the BioCog project (www.biocog.eu), which is an international, prospective, multicenter observational study that recruited patients at the anesthesiology departments of the Charité – Universitätsmedizin Berlin, in Germany, and the University Medical Center Utrecht in the Netherlands. The project was designed to identify biomarker panels associated with an increased risk of POD and POCD, as well as possible clinical outcome predictors [20]. The project was registered (ClinicalTrials.gov: NCT02265263), approved by Ethics Committees in both countries (ref.: EA2/092/14 and 14-469), and was conducted in accordance with the declaration of Helsinki Written. All relevant data protection regulations were followed, and informed consent was obtained from all patients.

The study included patients aged ≥ 65 years undergoing elective surgery with an expected surgical duration of at least 60 min, and a Mini-Mental-State-Examination (MMSE) score > 24 points. Detailed inclusion and exclusion criteria are described in a previous publication [20, 21]. Within the study, further publications with other questions have been published. Worth mentioning here seem the manuscripts, which investigate the association between preoperative medication use and development of POD and POCD [22] or between three Anticholinergic Drug Scales and development of POD [23] and between radiological, chemical and pharmacological cholinergic system parameters and preoperative neurocognitive disorder [21].

### Baseline measurements

Baseline measurements included: age, sex, Charlson Comorbidity Index (CCI) [24], duration of anesthesia and education according to the International Standard Classification of Education (ISCED).

### Postoperative delirium

POD was assessed daily through a validated delirium screening (using the Nursing Delirium Screening Scale, Confusion Assessment Method and a patient chart review) until the 7th postoperative day. For a detailed description of the assessment, please refer to previous publications [22, 23].

### Postoperative cognitive dysfunction (POCD)

A neuropsychological test battery was used to identify POCD, consisting of paper-based and computerized assessments performed prior to surgery and at a three-month follow-up. For a detailed description of the assessment, please refer to a previous publication [22].

### Genotyping

Blood samples were obtained from patients preoperatively. The DNA was extracted from whole blood or buffy coat with the ReliaPrep™ Blood gDNA Miniprep System (Promega GmbH, Walldorf, Germany) and with the Maxwell®RSC Buffy Coat DNA Kit (Promega GmbH) according to the manufacturer's instructions. For ReliaPrep™ System we adopted the protocol: Lysis 15 min at 56 °C, all steps for mixing at least 20 s. The Maxwell®RSC Kit was applied automatically using the Maxwell® RSC 48 (Promega GmbH). Genotyping was performed on the Illumina Infinium Global Screening Array (GSA) v2.0 (Illumina, Inc., San Diego, CA, USA) using a semiautomated protocol. All laboratory procedures were performed in accordance with the manufacturer's instructions. Illumina raw intensity files (.idat) were uploaded together with the Illumina GSA v2.0 manifest (.bmp) and cluster file (.egt) into the GenomeStudio v2.0 software and genotypes were subsequently exported to PLINK format. Afterwards, coordinates of genetic variants were converted according to the newest human genome build (hg38), as genetic variants were initially aligned to hg19. For this, we used an executable of the liftOver tool provided in the University of California Santa Cruz (UCSC) Genome Browser tool suite [25] and a wrapper script provided on GitHub [26] for files in PLINK-format.

Quality Control was performed in R software environment (version 3.5.1) [R Core Team (2017). R: A language and environment for statistical computing. R Foundation for Statistical Computing, Vienna, Austria. URL https://www.R-project.org/] and with PLINK 27 [Package: PLINK (v1.90b6.12), Author: Shaun Purcell, URL: http://pngu.mgh.harvard.edu/purcell/plink/] [27].

We filterd for chromosomes 1–22 and included them for further analysis. Therefore, we removed genetic variants with no chromosomal information, and variants that lay on mitochondrial chromosomes, on allosomes or on uncommon chromosome variants. Afterwards, we filtered out monomorphic variants that were not in Hardy–Weinberg Equilibrium (*p* < 0.0001). Lastly, we checked the Multidimensional scaling (MDS) plots for possible outliers (see Additional file 1: Figure S1, Additional file 2: Figure S2).

### Analysis

The baseline characteristics are shown as median with interquartile ranges, or frequencies with percentages. Group differences were tested using Mann–Whitney *U* test or Chi-Square Test, as appropriate.

### GWAS

After quality control, we first performed multiple logistic regression analysis with PLINK in a GWAS approach, with either POD or POCD as the response and each variant as the predictor, adjusting for possible confounding variables. We adjusted for variables that were chosen a priori and included age, CCI and duration of anesthesia for the regression analysis of POD, and age, sex, CCI, education (according to ISCED, with regard to level 1–4 which corresponds to a lower educational level) and duration of anesthesia for the regression analysis of POCD. We considered a *p*-value threshold of 5 × 10^− 8^ as genome-wide significant and 1 × 10^− 5^ as exploratory significant. The retrieved odds ratios associated with variants refer to minor allele counts (0, 1, 2). We visually checked the results by generating a quantile–quantile (Q-Q-plot) and calculating the inflation factor λ (see Additional file 3: Figure S3, Additional file 4: Figure S4).

### CGAS

Secondly we investigated genetic variants of cholinergic genes in a candidate gene approach. We have identified cholinergic genes of the cholinergic synapse according to the KEGG pathway [17–19]. These includes genes for: acetylcholinesterase (ACHE), choline acetyltransferase (CHAT), high affinity choline transporter (SLC5A7), vesicular acetylcholine transporter (SLC18A3), cholinergic receptor nicotinic alpha 3 subunit (CHRNA3), cholinergic receptor nicotinic alpha 4 subunit (CHRNA4), cholinergic receptor nicotinic alpha 6 subunit (CHRNA6), cholinergic receptor nicotinic alpha 7 subunit (CHRNA7), cholinergic receptor nicotinic beta 2 subunit (CHRNB2), cholinergic receptor nicotinic beta 4 subunit (CHRNB4), cholinergic receptor muscarinic 1 (CHRM1), cholinergic receptor muscarinic 2 (CHRM2), cholinergic receptor muscarinic 3 (CHRM3), cholinergic receptor muscarinic 4 (CHRM4) and cholinergic receptor muscarinic 5 (CHRM5). We retrieved the respective gene ranges including exons and introns from gene database of the National Center for Biotechnology Information [28].

Here we have applied the identical regression models with the same confounders as for the GWAS approach. For the CGAS, we assumed an exploratory significance level of 0.05 and liberally adjusted the *p*-values of the variants in each gene via Benjamini–Hochberg correction. Again, odds ratios associated with variants refer to minor allele counts. Statistical analyses were conducted with IBM© SPSS© Statistics, Version 23 [Copyright 1989, 2015 by SPSS Inc., Chicago, Illinois, USA], as well as R software environment (version 3.5.1) and PLINK 27 [27].

## Results

**Fig. 1:**
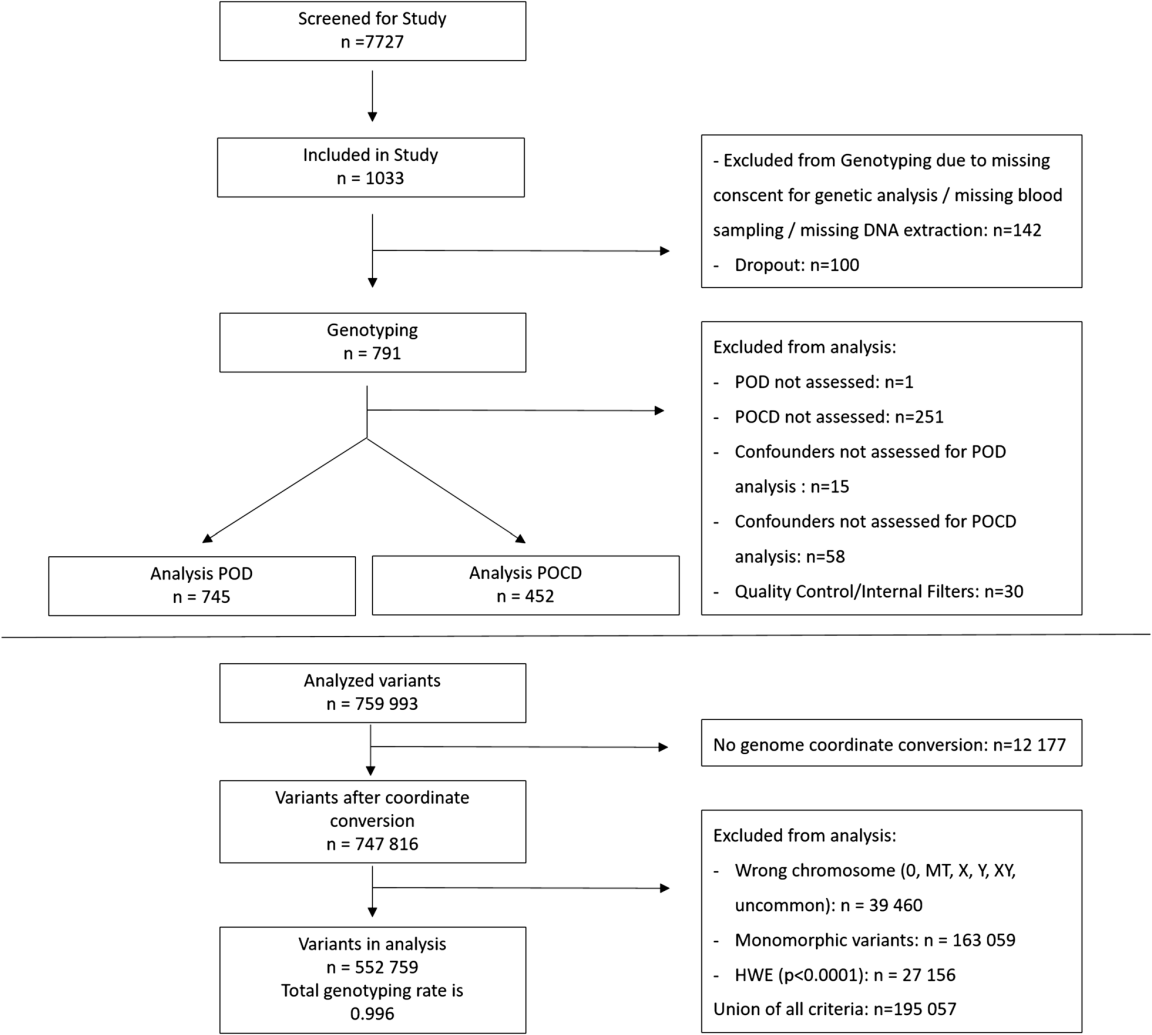
Flow chart

Between October 2014 and April 2017, a total of 1033 patients could be enrolled at the two study sites (Berlin, Germany and Utrecht, Netherlands). Following removal of drop-outs, loss to follow-up and missing data, 745 patients could ultimately be included in the POD analysis, and 452 in the POCD analysis (Fig. 1). The rate of POD within this group was 20.8% (155 patients), and the rate of POCD was 10.2% (46 patients) (see Tables 1 and 2). Patients developing POD were shown to be significantly older, have more comorbidities in terms of CCI scores, and longer duration of anesthesia.

**Table 1:** Patient characteristics for POD analysis (n = 745)

Characteristic	POD (n = 155) (20.8%)	No POD (n = 590) (79.2%)	*P*
	n = 745		
Age [years]	74 [70; 76]	71 [68; 75]	< 0.001^a^
Sex			
Female	73 (47.1%)	247 (41.9%)	0.24^b^
Charlson Comorbidity Index	1.84 ± 1.5	1.33 ± 1.5	< 0.001^a^
Duration of anesthesia [min]	305 [202; 470]	175 [107; 269]	< 0.001^a^

Data is shown as median [IQR 25; IQR 75], or as mean ± SD. Categorical data is shown as frequencies (percentages). Differences between patient groups with and without POD were evaluated with Mann–Whitney *U* test (a) or Chi-Square Test (b), whereas a *P* ≤ 0.05 was considered as statistically significant

IQR, interquartile range; SD, standard deviation

**Table 2:** Patient characteristics for POCD analysis (n = 452)

Characteristic	POCD (n = 46) (10.2%)	No POCD (n = 406) (89.8%)	*P*
	n = 452		
Age [years]	74 [70; 77]	71 [68; 75]	0.006^a^
Sex			
Female	26 (56.5%)	158 (38.9%)	0.02^b^
Charlson Comorbidity Index	1.74 ± 1.6	1.18 ± 1.4	0.006^a^
Duration of anesthesia (min)	202 [120; 308]	198 [121; 292]	0.99^a^

Data is shown as median [IQR 25; IQR 75], or as mean ± SD. Categorical data is shown as frequencies (percentages). Differences between patient groups with and without POD were evaluated with Mann–Whitney *U* test (a) or Chi-Square Test (b), whereas a *P* ≤ 0.05 was considered as statistically significant

IQR, interquartile range; SD, standard deviation

Similarly, patients that developed POCD were shown to be older and have higher CCI scores than those that did not, and although there were no differences in duration of anesthesia, there were differences in sex.

### GWAS

**Fig. 2:**
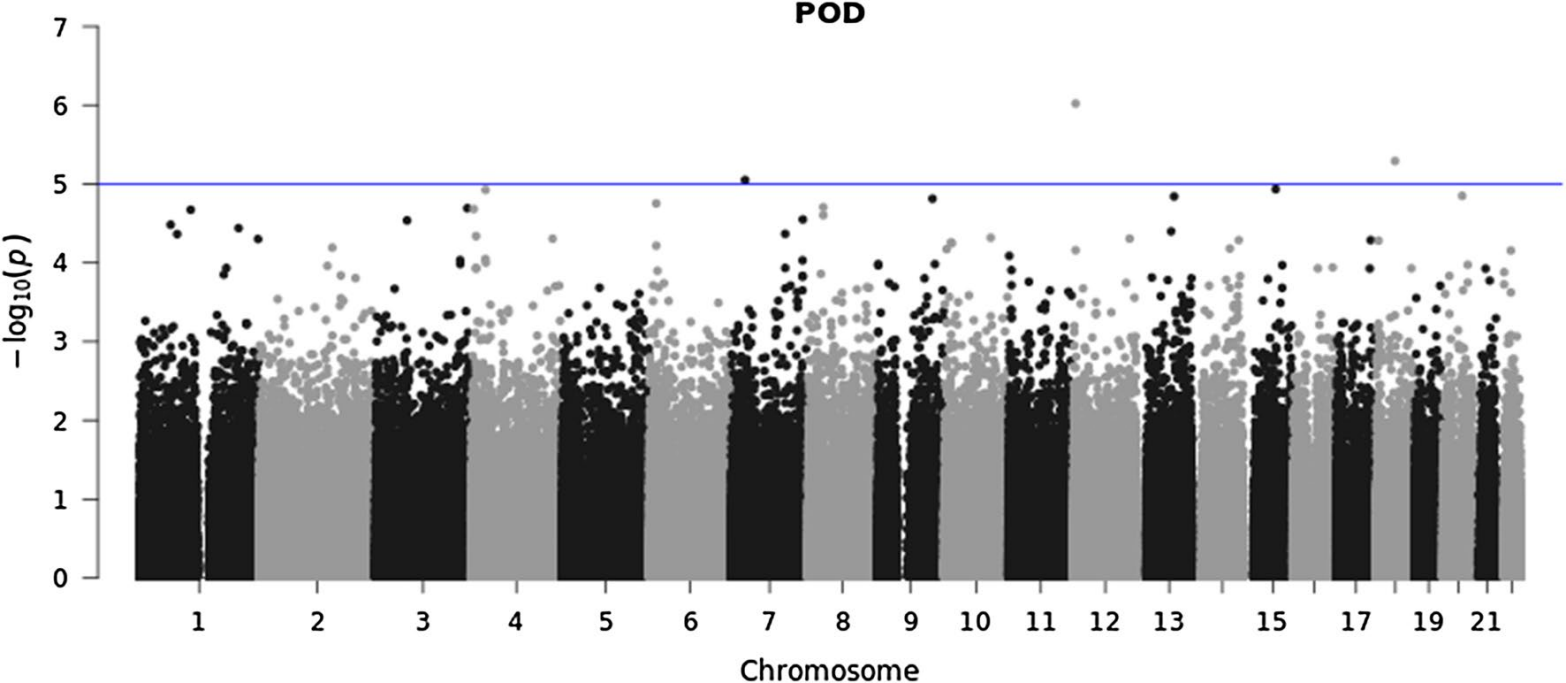
Manhattan plot for genome-wide association for POD (n = 745)

**Fig. 3:**
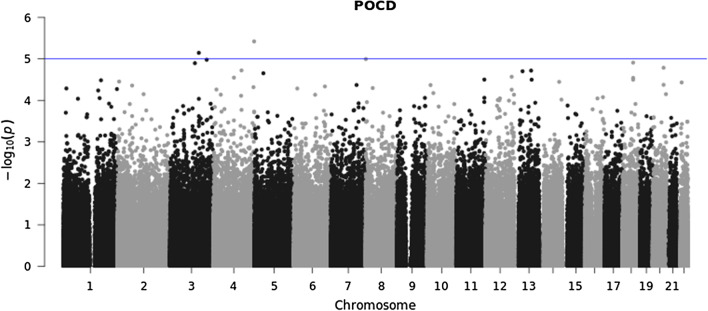
Manhattan plot for genome-wide association for POCD (n = 452)

747,816 variants were included in Quality Control (QC) and 552,759 variants passed QC. Therefore, in total 552,759 genetic variants were analyzed with a total genotyping rate of 0.996 (Fig. 1). In the GWAS approach no locus reached genome-wide significance (5 × 10^− 8^). When we applied the same level of significance as in a previous report of 1 × 10^− 5^ [14], we found three variants: rs12423672, rs75787432 and rs12155347 significantly associated with POD (see Fig. 2 and Additional file 5: Table S1). Additionally, two genetic variants: rs116044365 and rs73217998 were significantly associated with POCD (see Fig. 3 and Additional file 5: Table S2). Of these, only rs75787432 (Long Intergenic Non-Protein Coding RNA 669) and rs73217998 (acyl-CoA dehydrogenase family member 11) are located in gene regions.

### CGAS

In the candidate gene approach, we could identify three genetic variants of the cholinergic system associated with POD when considering confounding factors (Table 3).

**Table 3:** Overview over SNPs significantly associated with POD in CGAS approach

Chr	SNP	Gene name	Position	OR [95% CI]	*P*-value	Adjusted *P*-value
7	rs8191992	CHRM2	137,016,561	0.61 [0.46; 0.80]	< 0.001	0.01
7	rs6962027	CHRM2	137,017,188	1.59 [1.22; 2.09]	< 0.001	0.01
11	rs2067482	CHRM4	46,385,217	1.64 [1.10; 2.44]	0.01	0.03

Entered variables into logistic regression analysis: age (years), Charlson Comorbidity Index and duration of anesthesia. Data are expressed as odds ratio (OR) and 95% confidence interval (CI). OR refer to minor allele counts (0, 1, 2). *P*-values were adjusted for multiple testing by applying the Benjamini–Hochberg method according to the number of SNPs examined per cholinergic gene. Adjusted *P* ≤ 0.05 was considered as statistically significant

Chr, Chromosome; SNP, single-nucleotide polymorphism

The single-nucleotide polymorphism (SNP) rs8191992 of the CHRM2 gene was significantly associated with POD (Odds ratio (OR): minor allele carriers compared to non-carriers [95% confidence interval (CI)], 0.61 [0.46; 0.80], adjusted *p*-value = 0.01). rs8191992 is a 3′ prime UTR variant (T > A) with a European minor allele frequency (MAF) [29] of 0.46 (T). In addition, another SNP of the CHRM2 gene: rs6962027, which is a 3′ prime UTR variant, too (T > A/T > G) with a European MAF of 0.45 (T), was significantly associated with POD (OR [95% CI], 1.60 [1.22; 2.09], adjusted *p*-value = 0.01). Furthermore, rs2067482, a synonymous SNP (G > A) of the CHRM4 gene with a European MAF of 0.18 (A), was found to be significantly associated with POD (OR [95% CI], 1.64 [1.10; 2.44], adjusted *p*-value = 0.03). In our cohort the MAFs are comparable to the European MAFs: rs8191992 = 0.50 (T), rs6962027 = 0.50 (T) and rs2067482 = 0.18 (A).

In POCD analysis, we did not find any genetic variant of the cholinergic system to be significantly associated with POCD in the candidate gene approach.

## Discussion

The aim of this study was to investigate whether genetic variants in the cholinergic candidate genes are associated with POD or POCD in elderly patients, and found using a candidate gene approach three genetic variants of the cholinergic genes CHRM2 and CHRM4 to be associated with the development of a POD.

In the research field of delirium, there are so far only two studies that have conducted a GWAS [13, 14], whereas in the field of postoperative neurocognitive disorders (which includes POCD), to the best of our knowledge, no GWAS have yet been published. With regard to POD, a study by McCoy et al. was able to identify a single locus on Chr2 associated with the development of delirium in hospitalized patients [13]. It contained multiple genes, none of these, however, was part of the cholinergic system. The most important aspect to be taken into account in this study is, that delirium was solely defined by an electronic health record–based case definition. There is no information on routine assessment for delirium in the study center, so it is difficult to judge whether the evaluation of the electronic health record can lead to a valid evaluation of delirium incidence in the study population. The stated delirium incidence of 7.5%, may suggest that many delirious cases in this study could be falsely negatively assigned.

A study by Westphal et al. described two SNPs in long intergenic non-protein coding RNAs, which were associated not with genome-wide significance, but with a *p*-value of < 1 × 10^− 5^ to the development of POD in patients after non-emergent cardiac surgery [14]. In this study it should be noted that cardio surgical patients are extremely vulnerable to the development of POD, in contrast to our heterogeneous surgical cohort.

In our study, no locus reached genome-wide significance in the GWAS approach, neither for POD nor for POCD. Considering the constraints of the two previous studies: uncertain delirium assessment and failure to achieve genome-wide significance, our results are consistent with this. This can most likely be explained by the fact that these are multifactorial and most likely polygenic diseases, which are difficult to study in a GWAS approach. In addition, the number of participants, both in our study and in the cited ones, is very small for a GWAS approach. If there would be an associated locus with a much lower MAF, we might overlook it in our population due to the small number of cases. However, it is also possible that there is actually no connection or that the effect is too small to see a significant association in these extensive tests. Furthermore, we must take into account that there are factors other than genetic variants that may influence the expression of relevant genes, which could lead to the development of POD and POCD. For example, there are already studies suggesting that epigenetic regulation of mainly proinflammatory [30] and also neurotransmitter genes, especially cholinergic genes [31] may be involved in the development of delirium.

Under these considerations, it seemed reasonable to apply a candidate gene approach. In this approach we have referred to the cholinergic hypothesis. Hereby one supposes that cholinergic neurotransmission plays an important role in cognitive performance, and that cholinergic inhibition could suppress the formation of a circulus vitiosus, where neuroinflammation is maintained by the activation of microglia cells. Van Gool postulated that any dysfunction in cholinergic neurotransmission could hinder this mechanism and promote the development of delirium [12]. Since there is no evidence in the literature indicating which cholinergic genes or variants could be responsible for the disturbance of cholinergic neurotransmission, we decided to investigate all cholinergic genes involved in cholinergic neurotransmission according to the KEGG pathway [19].

In the candidate gene approach we were able to identify an association between genetic variants of the muscarinic cholinergic receptor genes CHRM2 and CHRM4 to be associated with the development of POD. In addition to CHRM1, CHRM2 and CHRM4 are among the most relevant receptors in the CNS. As G-protein-coupled receptors, they activate signaling pathways that are important for synaptic plasticity, the modulation of neuronal excitability, and the feedback regulation of Acetylcholine (Ach) release [32, 33]. The activation of both receptors terminates in the same pathway. Both feedback regulation and synaptic plasticity are transmitted by activating the guanine nucleotide-binding protein [19]. In addition, CHRM2 and CHRM4 receptors are also thought to be related to cognitive performance [34–36]. So far, no explicit connection with the development of delirium could be shown. Only in an Asian candidate gene study an association between the CHRM2 gene (rs1824024) and the development of delirium tremens, which is the severe form of alcohol withdrawal, was also shown [37]. Of particular interest was the finding that the genetic variant was not associated with alcohol dependence per se.

The genetic variants (rs8191992, rs6962027 and rs2067482) we have identified have also not been described in the literature in connection with POD and POCD, so far. For rs8191992 an association with intelligence quotient (IQ) [38, 39] and with cognitive flexibility (suppression of no longer relevant information and usage of prior information) [40] was merely described. In addition, rs8191992 was described to predict cardiac mortality after acute myocardial infarction and to determine cardiac function in a postexercise recovery phase [41]. Consistent with this, in another study investigating patients with schizophrenia, it was shown that rs8191992 was associated with decreased activity of the autonomic nervous system [42]. In addition, it was described that rs8191992 has an impact on visual attention when it acts synergistically with another variant of the nicotinic receptor CHRNA4 [43]. In contrast, other studies on cognitive function could not show any impact of rs8191992 [44, 45]. rs6962027 in turn was found to be associated with asthma susceptibility [46] and with poor bronchodilator response in asthmatic patients [47]. And rs2067482 was described to be associated with schizophrenia susceptibility [48].

### Strengths and limitations

Important strengths of this study include the prospective multicenter design, as well as the rigorous assessment of POD and POCD. The POCD neuropsychological test battery took into account several cognitive domains, and followed a validated standard with limited rater effects. The R algorithm employed is freely available, allowing comparability of results with other major investigations [49].

However, there are important limitations to this study. One of the major limitations is that with the achieved sample size, GWAS are usually not performed. Nonetheless, given the scarcity of studies and our unique database, we performed a GWAS in an exploratory manner. Furhermore, the incidence of POCD was low (10%), which is likely to limit statistical power. Although many patients were ultimately excluded from the analyses, the incidence of POD and POCD did not differ between the enrolled and analyzed collectives, so that this limitation is not expected to alter the results. Likewise, although patients with POCD tended to have slightly lower CCI scores, the remaining patient characteristics did not differ significantly after patient exclusion. The results were not corrected for multiple testing, as there are no uniform guidelines as to how multiple testing can be considered within CGAS. We decided to consider how many genetic variants were studied per candidate gene. Against this background, the adjusted *p*-values should not be interpreted as absolute values, but as an orientation to order the impact of the different genetic variants.

## Conclusion

In conclusion, we found an association between genetic variants of CHRM2 and CHRM4 and the development of POD in a candidate gene approach. Our results are in agreement with the hypothesis that cholinergic neurotransmission and synaptic plasticity are involved in POD. Further studies are needed to investigate these hypotheses.

## Supplementary Information

**Additional file 1: Figure S1**. Multidimensional Scaling (MDS) Plots for the detection of outliers in POD analysis (n = 745). Different colourscales indicate disease status POD (0 = No POD, 1 = POD). No individuals were removed. (A–F) indicate different components in comparison.

**Additional file 2: Figure S2**. Multidimensional Scaling (MDS) Plots for the detection of outliers in POCD analysis (n = 452). Different colourscales indicate disease status POD (0 = No POD, 1 = POD). No individuals were removed. (A–F) indicate different components in comparison.

**Additional file 3: Figure S3**. Quantile–Quantile-Plot (Q-Q-Plot) of genome-wide association results for POD (n = 745).

**Additional file 4: Figure S4**. Quantile–Quantile-Plot (Q-Q-Plot) of genome-wide association results for POCD (n = 452).

**Additional file 5: Table S1**. Overview over SNPs exploratively associated with POD in GWAS approach. **Table S2**. Overview over SNPs exploratively associated with POCD in GWAS approach. **Table S3**. Overview of the total number of SNPs examined per cholinergic gene

## Data Availability

Since consent for publication of datasets generated and analyzed during the current study has not been given and cannot be obtained since some study participants have already passed away, data are not publicly available but are available from the corresponding author (claudia.spies@charite.de) on reasonable request. This procedure is necessary due to the National General Data Protection Regulation (DS-GVO).

## References

[1] American Psychiatric Association (2013). Diagnostic and statistical manual of mental disorders.

[2] Aldecoa C, Bettelli G, Bilotta F, Sanders RD, Audisio R, Borozdina A (2017). European Society of Anaesthesiology evidence-based and consensus-based guideline on postoperative delirium. Eur J Anaesthesiol.

[3] Moskowitz EE, Overbey DM, Jones TS, Jones EL, Arcomano TR, Moore JT (2017). Post-operative delirium is associated with increased 5-year mortality. Am J Surg.

[4] Davis DH, Muniz Terrera G, Keage H, Rahkonen T, Oinas M, Matthews FE (2012). Delirium is a strong risk factor for dementia in the oldest-old: a population-based cohort study. Brain J Neurol.

[5] Inouye SK, Marcantonio ER, Kosar CM, Tommet D, Schmitt EM, Travison TG (2016). The short-term and long-term relationship between delirium and cognitive trajectory in older surgical patients. Alzheimer's Dement J Alzheimer's Assoc.

[6] Inouye SK (1999). Predisposing and precipitating factors for delirium in hospitalized older patients. Dement Geriatr Cogn Disord.

[7] Wang J, Li Z, Yu Y, Li B, Shao G, Wang Q (2015). Risk factors contributing to postoperative delirium in geriatric patients postorthopedic surgery. Asia-Pac Psychiatry Off J Pac Rim Coll Psychiatr.

[8] Dunne SS, Coffey JC, Konje S, Gasior S, Clancy CC, Gulati G (2021). Biomarkers in delirium: a systematic review. J Psychosom Res.

[9] van Munster BC, de Rooij SE, Korevaar JC (2009). The role of genetics in delirium in the elderly patient. Dement Geriatr Cogn Disord.

[10] Sepulveda E, Adamis D, Franco JG, Meagher D, Aranda S, Vilella E (2021). The complex interaction of genetics and delirium: a systematic review and meta-analysis. Eur Arch Psychiatry Clin Neurosci.

[11] Maldonado JR (2017). Acute brain failure: pathophysiology, diagnosis, management, and sequelae of delirium. Crit Care Clin.

[12] van Gool WA, van de Beek D, Eikelenboom P (2010). Systemic infection and delirium: when cytokines and acetylcholine collide. Lancet (Lond, Engl).

[13] McCoy TH, Hart K, Pellegrini A, Perlis RH (2018). Genome-wide association identifies a novel locus for delirium risk. Neurobiol Aging.

[14] Westphal S, Stoppe C, Gruenewald M, Bein B, Renner J, Cremer J (2019). Genome-wide association study of myocardial infarction, atrial fibrillation, acute stroke, acute kidney injury and delirium after cardiac surgery—a sub-analysis of the RIPHeart-Study. BMC Cardiovasc Disord.

[15] Goodall R (2004). Cholinesterase: phenotyping and genotyping. Ann Clin Biochem.

[16] Shenhar-Tsarfaty S, Berliner S, Bornstein NM, Soreq H (2014). Cholinesterases as biomarkers for parasympathetic dysfunction and inflammation-related disease. J Mol Neurosci MN.

[17] Kanehisa M, Goto S (2000). KEGG: Kyoto encyclopedia of genes and genomes. Nucleic Acids Res.

[18] Kanehisa M, Sato Y, Furumichi M, Morishima K, Tanabe M (2019). New approach for understanding genome variations in KEGG. Nucleic Acids Res.

[19] KEGG. Cholinergic synapse—Reference pathway 2020. https://www.kegg.jp/kegg-bin/highlight_pathway?scale=1.0&map=map04725&keyword=cholinergic.

[20] Winterer G, Androsova G, Bender O, Boraschi D, Borchers F, Dschietzig TB (2018). Personalized risk prediction of postoperative cognitive impairment—rationale for the EU-funded BioCog project. Eur Psychiatry J Assoc Eur Psychiatr.

[21] Heinrich M, Müller A, Lammers-Lietz F, Borchers F, Mörgeli R, Kruppa J (2020). Radiological, chemical and pharmacological cholinergic system parameters and neurocognitive disorders in older pre-surgical adults. J Gerontol A Biol Sci Med Sci.

[22] Heinrich M, Nottbrock A, Borchers F, Mörgeli R, Kruppa J, Winterer G (2021). Preoperative medication use and development of postoperative delirium and cognitive dysfunction. Clin Transl Sci.

[23] Heinrich M, Müller A, Cvijan A, Mörgeli R, Kruppa J, Winterer G (2021). Preoperative comparison of three anticholinergic drug scales in older adult patients and development of postoperative delirium: a prospective observational study. Drugs Aging.

[24] Charlson ME, Pompei P, Ales KL, MacKenzie CR (1987). A new method of classifying prognostic comorbidity in longitudinal studies: development and validation. J Chronic Dis.

[25] (UCSC) UoCSC. Lift Genome Annotations. https://genome.ucsc.edu/cgi-bin/hgLiftOver.

[26] (UCSC) UoCSC. liftOverPlink. https://github.com/sritchie73/liftOverPlink.

[27] Purcell S, Neale B, Todd-Brown K, Thomas L, Ferreira MA, Bender D (2007). PLINK: a tool set for whole-genome association and population-based linkage analyses. Am J Hum Genet.

[28] Bethesda (MD): National Library of Medicine (US) NCfBI. Gene [Internet]. https://www.ncbi.nlm.nih.gov/gene/ (2004).

[29] Phan YJL, Zhang H, Qiang W, Shekhtman E, Shao D, Revoe D, Villamarin R, Ivanchenko E, Kimura M, Wang ZY, Hao L, Sharopova N, Bihan M, Sturcke A, Lee M, Popova N, Wu W, Bastiani C, Ward M, Holmes JB, Lyoshin V, Kaur K, Moyer E, Feolo M, Kattman BL. "ALFA: Allele Frequency Aggregator." National Center for Biotechnology Information, U.S. National Library of Medicine. www.ncbi.nlm.nih.gov/snp/docs/gsr/alfa/ (2020).

[30] Shinozaki G, Braun PR, Hing BWQ, Ratanatharathorn A, Klisares MJ, Duncan GN (2018). Epigenetics of delirium and aging: potential role of DNA methylation change on cytokine genes in glia and blood along with aging. Front Aging Neurosci.

[31] Saito T, Toda H, Duncan GN, Jellison SS, Yu T, Klisares MJ (2020). Epigenetics of neuroinflammation: immune response, inflammatory response and cholinergic synaptic involvement evidenced by genome-wide DNA methylation analysis of delirious inpatients. J Psychiatr Res.

[32] Volpicelli LA, Levey AI (2004). Muscarinic acetylcholine receptor subtypes in cerebral cortex and hippocampus. Prog Brain Res.

[33] Lebois EP, Thorn C, Edgerton JR, Popiolek M, Xi S (2018). Muscarinic receptor subtype distribution in the central nervous system and relevance to aging and Alzheimer's disease. Neuropharmacology.

[34] Seeger T, Fedorova I, Zheng F, Miyakawa T, Koustova E, Gomeza J (2004). M2 muscarinic acetylcholine receptor knock-out mice show deficits in behavioral flexibility, working memory, and hippocampal plasticity. J Neurosci.

[35] Carey GJ, Billard W, Binch H, Cohen-Williams M, Crosby G, Grzelak M (2001). SCH 57790, a selective muscarinic M(2) receptor antagonist, releases acetylcholine and produces cognitive enhancement in laboratory animals. Eur J Pharmacol.

[36] Popiolek M, Mandelblat-Cerf Y, Young D, Garst-Orozco J, Lotarski SM, Stark E (2019). In vivo modulation of hippocampal excitability by M4 muscarinic acetylcholine receptor activator: implications for treatment of Alzheimer's disease and schizophrenic patients. ACS Chem Neurosci.

[37] Malhotra S, Basu D, Ghosh A, Khullar M, Chugh N, Kakkar N (2018). An exploratory study of candidate gene(s) for Delirium Tremens: adding the new cholinergic dimension to the conundrum. Asian J Psychiatr.

[38] Comings DE, Wu S, Rostamkhani M, McGue M, Lacono WG, Cheng LS (2003). Role of the cholinergic muscarinic 2 receptor (CHRM2) gene in cognition. Mol Psychiatry.

[39] Dick DM, Aliev F, Kramer J, Wang JC, Hinrichs A, Bertelsen S (2007). Association of CHRM2 with IQ: converging evidence for a gene influencing intelligence. Behav Genet.

[40] Zink N, Bensmann W, Arning L, Stock AK, Beste C (2019). CHRM2 genotype affects inhibitory control mechanisms during cognitive flexibility. Mol Neurobiol.

[41] Hautala AJ, Tulppo MP, Kiviniemi AM, Rankinen T, Bouchard C, Mäkikallio TH (2009). Acetylcholine receptor M2 gene variants, heart rate recovery, and risk of cardiac death after an acute myocardial infarction. Ann Med.

[42] Miyauchi M, Kishida I, Suda A, Shiraishi Y, Hattori S, Fujibayashi M (2016). Association of the cholinergic muscarinic M2 receptor with autonomic nervous system activity in patients with schizophrenia on high-dose antipsychotics. Neuropsychobiology.

[43] Greenwood PM, Lin MK, Sundararajan R, Fryxell KJ, Parasuraman R (2009). Synergistic effects of genetic variation in nicotinic and muscarinic receptors on visual attention but not working memory. Proc Natl Acad Sci U S A.

[44] Lind PA, Luciano M, Horan MA, Marioni RE, Wright MJ, Bates TC (2009). No association between Cholinergic Muscarinic Receptor 2 (CHRM2) genetic variation and cognitive abilities in three independent samples. Behav Genet.

[45] Harris SE, Fox H, Wright AF, Hayward C, Starr JM, Whalley LJ (2007). A genetic association analysis of cognitive ability and cognitive ageing using 325 markers for 109 genes associated with oxidative stress or cognition. BMC Genet.

[46] Jiménez-Morales S, Jiménez-Ruíz JL, Del Río-Navarro BE, Navarro-Olivos E, Escamilla-Guerrero G, Savan R (2014). CHRM2 but not CHRM1 or CHRM3 polymorphisms are associated with asthma susceptibility in Mexican patients. Mol Biol Rep.

[47] Szczepankiewicz A, Breborowicz A, Sobkowiak P, Kramer L, Popiel A (2009). Association of A/T polymorphism of the CHRM2 gene with bronchodilator response to ipratropium bromide in asthmatic children. Pneumonol Alergol Pol.

[48] Scarr E, Um JY, Cowie TF, Dean B (2013). Cholinergic muscarinic M4 receptor gene polymorphisms: a potential risk factor and pharmacogenomic marker for schizophrenia. Schizophr Res.

[49] Spies CD, Knaak C, Mertens M, Brockhaus WR, Shadenok A, Wiebach J (2021). Physostigmine for prevention of postoperative delirium and long-term cognitive dysfunction in liver surgery: a double-blinded randomised controlled trial. Eur J Anaesthesiol.

