# Supplementary figures and images for: Association between genetic variants of the cholinergic system and postoperative delirium and cognitive dysfunction in elderly patients
Source: BMC Med Genomics. 2021 Oct 21;14:248. doi: 10.1186/s12920-021-01071-1 (PMC8529799; doi:10.1186/s12920-021-01071-1)

# MDS plots of SNPs for POD

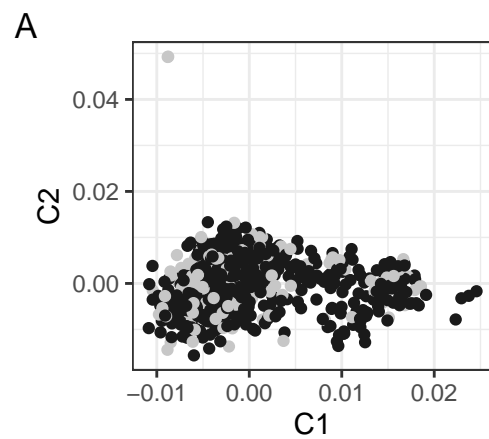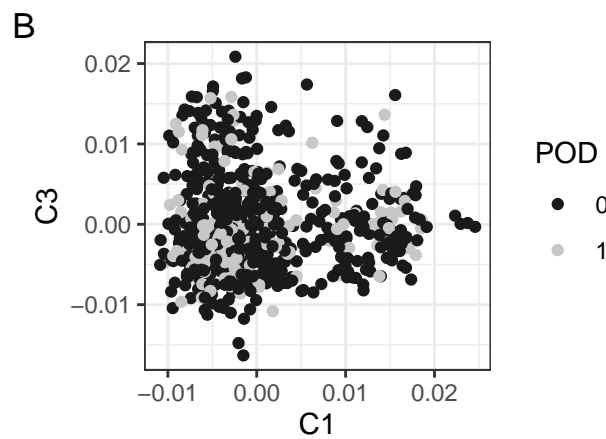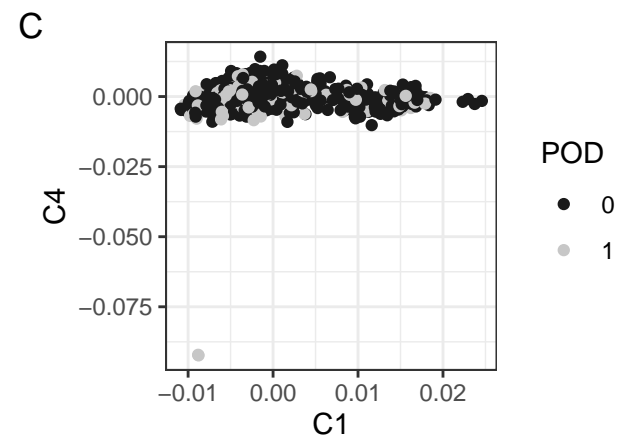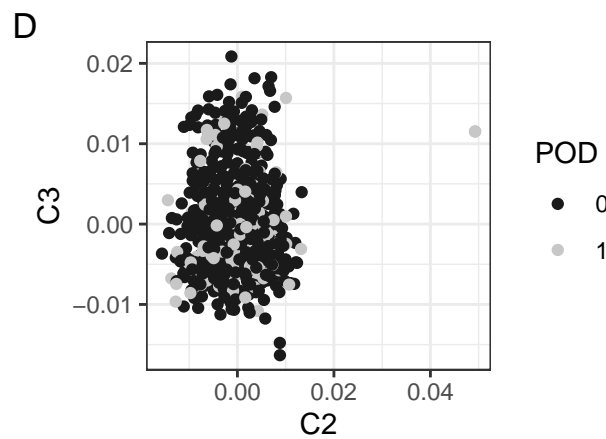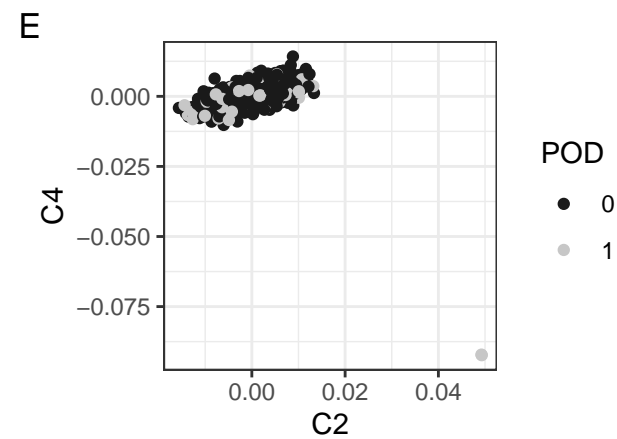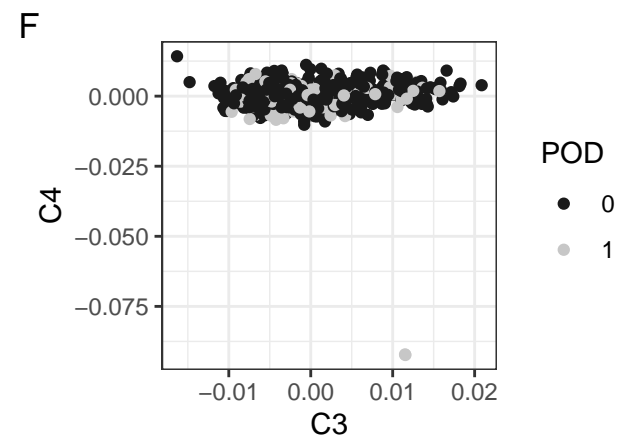

Supplement: Supplementary file 1 — Additional file 1: Figure S1. Multidimensional Scaling (MDS) Plots for the detection of outliers in POD analysis (n = 745). Different colourscales indicate disease status POD (0 = No POD, 1 = POD). No individuals were removed. (A–F) indicate different components in comparison. [file 12920_2021_1071_MOESM1_ESM.pdf]

# MDS plots of SNPs for POCD

A

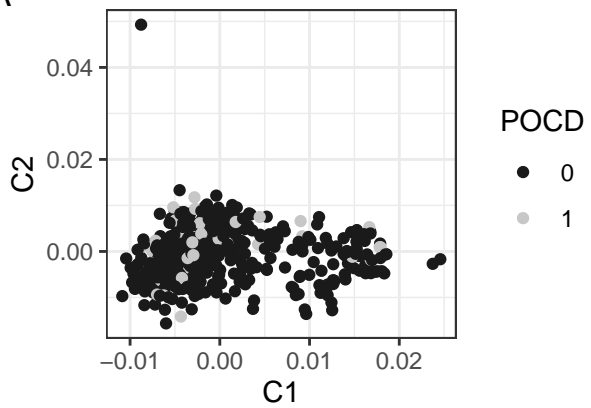

B

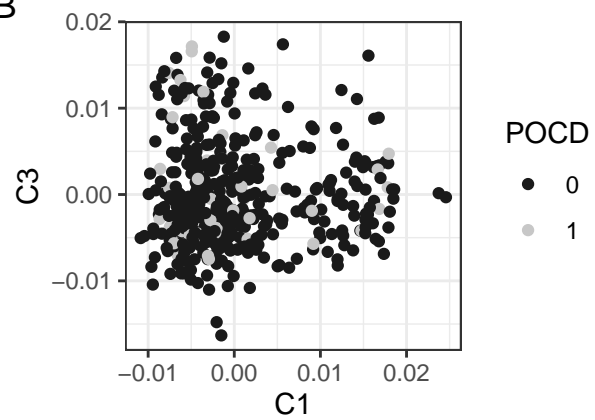

C

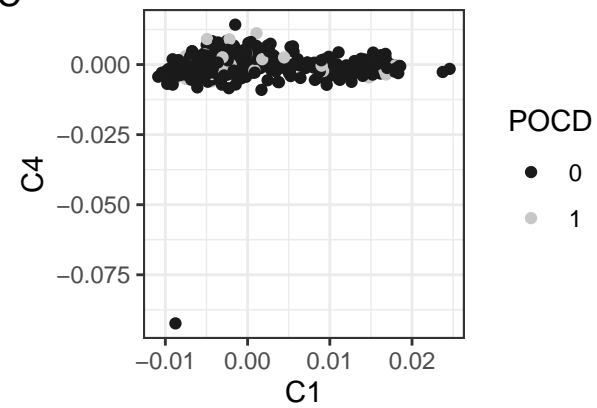

D

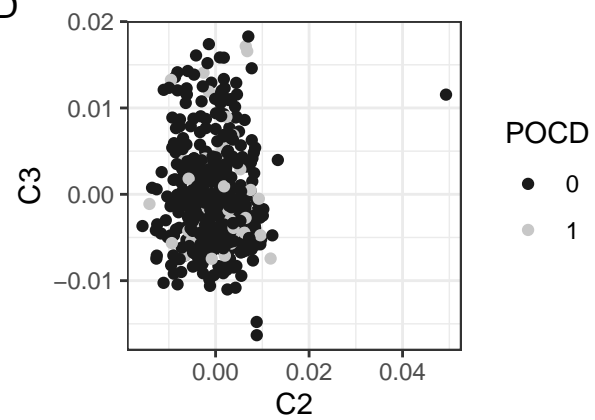

E

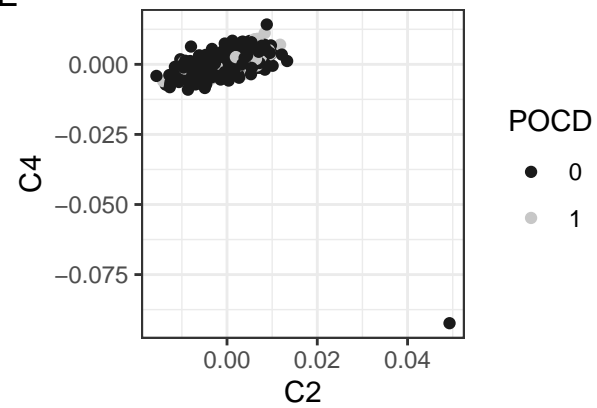

F

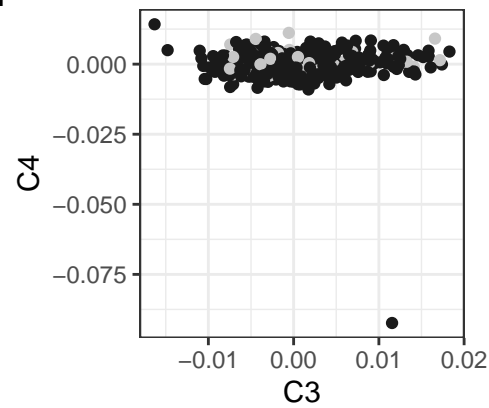

Supplement: Supplementary file 2 — Additional file 2: Figure S2. Multidimensional Scaling (MDS) Plots for the detection of outliers in POCD analysis (n = 452). Different colourscales indicate disease status POD (0 = No POD, 1 = POD). No individuals were removed. (A–F) indicate different components in comparison. [file 12920_2021_1071_MOESM2_ESM.pdf]

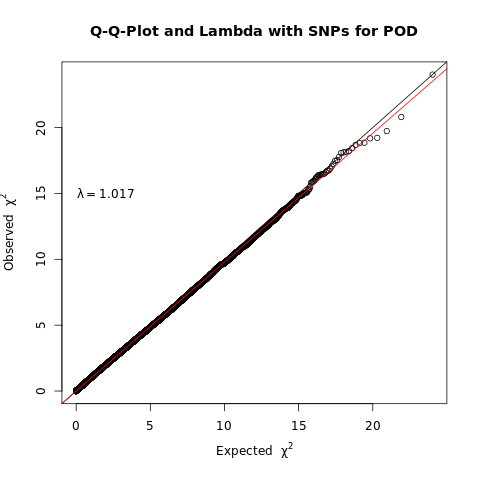

Supplement: Supplementary file 3 — Additional file 3: Figure S3. Quantile–Quantile-Plot (Q-Q-Plot) of genome-wide association results for POD (n = 745). [file 12920_2021_1071_MOESM3_ESM.png]

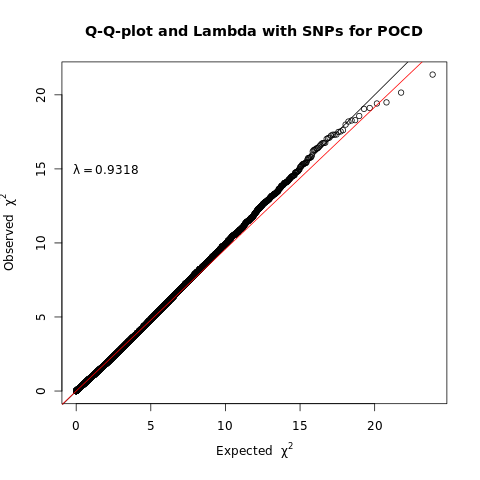

Supplement: Supplementary file 4 — Additional file 4: Figure S4. Quantile–Quantile-Plot (Q-Q-Plot) of genome-wide association results for POCD (n = 452). [file 12920_2021_1071_MOESM4_ESM.png]
