# Supplementary material for: Association between genetic variants of the cholinergic system and postoperative delirium and cognitive dysfunction in elderly patients
Source: BMC Med Genomics. 2021 Oct 21;14:248. doi: 10.1186/s12920-021-01071-1 (PMC8529799; doi:10.1186/s12920-021-01071-1)
Supplement: Supplementary file 5 — Additional file 5: Table S1. Overview over SNPs exploratively associated with POD in GWAS approach. Table S2. Overview over SNPs exploratively associated with POCD in GWAS approach. Table S3. Overview of the total number of SNPs examined per cholinergic gene [file 12920_2021_1071_MOESM5_ESM.docx]

**Additional file 5: Table S1.** **Overview over SNPs exploratively associated with POD in GWAS approach.**

Entered Variables into logistic regression analysis: Age (years), Charlson Comorbidity Index and duration of anesthesia. Data are expressed as Odds Ratio (OR) and 95%- Confidence Interval (CI). OR refer to minor allele counts (0, 1, 2). P ≤ 1 × 10− 5 was considered as statistically significant.

Chr –Chromosome, SNP - single-nucleotide polymorphism

| **Chr** | **SNP** | **Gene Name** | **Position** | **OR [95%-CI]** | **P-value** |
| --- | --- | --- | --- | --- | --- |
| 12 | rs12423672 | none | 4938539 | 2.79 [1.85; 4.21] | 0.10 × 10^− 5^ |
| 18 | rs75787432 | MIR924HG | 39632746 | 6.01 [2.78;13.00] | 0.51 × 10^− 5^ |
| 7 | rs12155347 | none | 28840164 | 4.57 [2.34;8.95] | 0.89 × 10^− 5^ |

**Additional file 5: Table S2. Overview over SNPs exploratively associated with POCD in GWAS approach.**

Entered Variables into logistic regression analysis: Age (years), Sex, Charlson Comorbidity Index and education (according to ISCED, with regard to level 1-4 which corresponds to a lower educational level). Data are expressed as Odds Ratio (OR) and 95%- Confidence Interval (CI). OR refer to minor allele counts (0, 1, 2). P ≤ 1 × 10− 5 was considered as statistically significant.

Chr –Chromosome, SNP - single-nucleotide polymorphism

| **Chr** | **SNP** | **Gene Name** | **Position** | **OR [95%-CI]** | **P-value** |
| --- | --- | --- | --- | --- | --- |
| 4 | rs116044365 | none | 188637992 | 13.96 [4.57;42.69] | 0.38× 10^− 5^ |
| 3 | rs73217998 | ACAD11 | 132566087 | 9.05 [3.46;23.66] | 0.72 × 10^− 5^ |

**Additional file 5: Table S3. Overview of the total number of SNPs examined per cholinergic gene**

SNP - single-nucleotide polymorphism

| **Gene Name** | **Number of SNPs** |
| --- | --- |
| ACHE | 2 |
| CHAT | 39 |
| SLC5A7 | 10 |
| SLC18A3 | 0 |
| CHRNA3 | 15 |
| CHRNA4 | 19 |
| CHRNA6 | 6 |
| CHRNA7 | 27 |
| CHRNB2 | 6 |
| CHRNB4 | 15 |
| CHRM1 | 4 |
| CHRM2 | 34 |
| CHRM3 | 107 |
| CHRM4 | 2 |
| CHRM5 | 6 |
